# MicroRNA-122-5p ameliorates tubular injury in diabetic nephropathy via FIH-1/HIF-1α pathway

**DOI:** 10.1080/0886022X.2022.2039194

**Published:** 2022-02-15

**Authors:** Li Cheng, Xinying Qiu, Liyu He, Li Liu

**Affiliations:** Department of Nephrology, The Second Xiangya Hospital at Central South University, Changsha, China

**Keywords:** MicroRNA-122-5p, tubular injury, diabetic nephropathy, FIH-1, HIF-1

## Abstract

Diabetes kidney disease (DKD) affects approximately one-third of diabetes patients, however, the specific molecular mechanism of DKD remains unclear, and there is still a lack of effective therapies. Here, we demonstrated a significant increase of microRNA-122-5p (miR-122-5p) in renal tubular cells in STZ induced diabetic nephropathy (DN) mice. Moreover, inhibition of miR-122-5p led to increased cell death and serve tubular injury and promoted DN progression following STZ treatment in mice, whereas supplementation of miR-122-5p mimic had kidney protective effects in this model. In addition, miR-122-5p suppressed the expression of factor inhibiting hypoxia-inducible factor-1 (FIH-1) *in vitro* models of DN. microRNA target reporter assay further verified FIH-1 as a direct target of miR-122-5p. Generally, FIH-1 inhibits the activity of HIF-1α. Our *in vitro* study further indicated that overexpression of HIF-1α by transfection of HIF-1α plasmid reduced tubular cell death, suggesting a protective role of HIF-1α in DN. Collectively, these findings may unveil a novel miR-122-5p/FIH-1/HIF-1α pathway which can attenuate the DN progression.

## Introduction

Diabetic kidney disease (DKD) is the leading cause of end-stage renal disease (ESRD) worldwide [1,2]. In addition, DKD also is the leading cause of morbidity and mortality in individuals with diabetes [3]. Generally, DKD is characterized by glomerular hypertrophy, proteinuria, decreased glomerular filtration, and renal fibrosis resulting in the loss of renal function [1]. Accordingly, more than 1/2 of patients with type 2 diabetes and 1/3 of those with type 1 diabetes develop DKD, and DKD is a prime reason for dialysis in many developed countries [4]. Thus, DKD poses a significant economic and health burden to the world. The pathogenesis of DKD is complex and apparently multifactorial, involving inflammation, hypoxia, oxidative stress, and apoptosis [5]. In addition, many new signaling molecules that regulate kidney fibrosis have been found, such as protective nature of endothelial glucocorticoid receptors, endothelial SIRT3, endothelial FGFR1 against renal fibrosis and, podocyte–glucocorticoid receptor signaling in protecting diabetic nephropathy [6–9]. Besides, some potential drugs have been identified in protecting DN, such as DPP-4 inhibitor linagliptin, empagliflozin, JAK-stat3 inhibitors, glycolysis inhibitors, ROCK inhibitors, mineralocorticoid antagonists, and ACE inhibitors [10–12]. Recently, a study has indicated that probucol could ameliorate EMT and lung fibrosis through restoration of SIRT3 expression. Thus, endothelial SIRT3 also could be the potential drug for DKD [13]. However, despite these progresses, the mechanisms of DN remain largely unclear, and effective treatment are still not available.

Usually, glomerular damage is considered the main pathological feature of DKD [14]. However, studies have shown that tubular injury is critical in DKD, and the degree of renal tubular injury is closely related to renal function [15]. A large number of studies have indicated that proximal tubule is uniquely susceptible to a variety of metabolic and hemodynamic factors associated with diabetes [15]. Thus, proximal tubule may be a new therapeutic target for patients with DKD. In addition, it has been indicated that tubular injury was a critical component of the early course of DKD and also suggested to contribute in a primary way, rather than a secondary manner, to the development of early DKD [16].

MicroRNAs (miRNAs) are a group of small non-coding RNA molecules composing of approximate 22 nucleotides. In mammalian cells, miRNAs repress gene expression mainly by binding to the 3′-untranslated regions (UTR) of their target gene mRNA, thereby blocking their translation [17]. Accumulating studies suggest that the majority of genes are subjected to miRNA regulation. Moreover, a single microRNA may regulate different genes and different miRNAs can regulate the same gene [18]. Till now, 39,000 miRNAs have been identified in humans, 365 of which are present on the renal cortex [19]. In DKD, alterations in miRNA expression influence the epithelial-to-mesenchymal transition (EMT) and endothelial-to-mesenchymal transition (EndMT) programs [20]. In the epithelial cells and endothelial cells, the antifibrotic miRNAs (such as miR-29 and let-7s) [21–23] and profibrotic microRNAs (such as miR-21 and miR-433) [24,25] are expressed physiologically. The differential expressions of microRNAs in the epithelial cells and endothelial cells regulate the biological pathways and signaling events and maintain the homeostasis. However, when healthy cells undergo the mesenchymal transition process, the expression of anti-fibrotic microRNAs decreases, while pro-fibrotic microRNA expression increases and disrupts the cellular homeostasis. In addition, miR-29 and miR-let-7s show crosstalk regulation by inducing FGFR1 phosphorylation and targeting TGFβR1. FGFR1 phosphorylation is critical for miR-let-7 production. In the presence of higher DPP-4 activity level or absence of AcSDKP, miR-let-7 families are down-regulated, which in turn causes activation of TGFβ signaling. Higher levels of TGFβ signaling results in suppression of miR-29 family expression and finally influences EndMT and fibrogenesis [23,26–28]. Taking together, these studied showed miRNAs were involved in the pathogenesis of tubular injury in DKD.

In the present study, we have demonstrated that the miR-122-5p was up-regulated in renal tubular cells in diabetic nephropathy (DN) mice. Functionally, miR-122-5p alleviated tubular cell death and kidney injury, finally affording a protective effect in DN. In contrast, suppression of miR-122-5p would aggravate kidney damage. Interestingly, we further identified FIH-1 (factor inhibiting hypoxia-inducible factor-1) was a direct target of miR-122-5p. Collectively, our study indicates that miR-122-5p could ameliorate tubular injury in diabetic nephropathy *via* FIH-1/HIF-1α pathway.

## Materials and methods

### Antibodies and special reagents

Antibodies: anti-FIH-1(ab237544) was from Abcam (Cambridge, UK). Anti-cleaved caspase-3(9664) and anti-GAPDH (5174) were from Cell Signaling Technology (Boston, MA). All secondary antibodies for immunoblotting analysis were from Thermo Fisher Scientific (Waltham, MA). FITC -conjugated goat anti-rabbit IgG were from Abcam. Special reagents: Digoxigenin-labeled mmu-miR-122-5p LNA probe (Servicebio, Wuhan, China), Fluorescence *in situ* hybridization Kit (Servicebio, Wuhan, China), miR-122-5p mimic (Ruibo, Guangzhou, China), anti-miR-122-5p (Ruibo, Guangzhou, China), Lipofectamine 2000 (Thermo Fisher, Waltham, MA).

### Animals and DN induction

Eight-week-old male C57BL/6 mice were purchased from the Slaccas Animal Laboratory (Changsha, China) and housed under controlled environmental conditions (temperature of 22C, 12 h darkness period). The protocol was approved by the Institutional Animal Care and Use Committee. DN was induced by STZ (Sigma-Aldrich, St. Louis, MO) injection intraperitoneally. For STZ induction of diabetes, mice at 4 weeks of age were injected with 50 mg/kg body weight STZ for 5 consecutive days according to a standard protocol [29]. Animals with >250 mg/dl fasting blood glucose for two consecutive readings were considered diabetic. Control mice were injected with normal saline. The mice were euthanized after 12 weeks. In some experiments, miR-122-5p mimic (3mg/kg), anti-miR-122-5p LNA (6 mg/kg), or NC oligonucleotide LNA were delivered to mice through tail vein injection every 2 week after STZ injection [30].

### Morphological studies

Kidney tissues were fixed with 4% buffered paraformaldehyde and were embedded in paraffin; 4-μm-thick sections were subsequently prepared. The sections were then subjected to hematoxylin-eosin (HE), periodic acid-Schiff (PAS) and Masson staining. Quantification of renal tubular atrophy was performed in a blinded manner and scored by the percentage of atrophied tubules: 0, no damage; 1, <25%; 2, 25–50%; 3, 50–75%; 4, >75% [31].

### Analysis of metabolic and physiological parameters

The body weight and blood glucose level were measured every week, and urine was collected before euthanasia. Urine *N*-acetyl-β-d glucosaminidase (NAG) was assessed using an automated colorimetric method (Pacific). Urinary creatinine and albumin were measured with a creatinine assay kit and an Albuwell M kit (Exocell) [32].

### Fluorescence *In Situ* Hybridization

Fluorescence *in situ* hybridization (FISH) was performed according to the manufacturer's instructions. Briefly, kidneys were harvested from control and STZ-treated mice to prepare 4-micron paraffin section. The sections were treated with 20 μg/ml proteinase K for permeabilization, and then incubated with pre-hybridization solution at 78 °C for 1 h. Remove pre-hybridization solution and add digoxigenin-labeled mmu-miR-122-5p LNA probe over night at 37 °C. At the second day, after wash, bovine serum albumin (BSA) was added for blocking. Then the anti-digoxigenin-HRP was used at 37 °C for 1 h. CY3-TSA and DAPI assay were used to indicate the positive areas and cell nucleus respectively. The images were acquired from a fluorescence microscope and the representative figures were exhibited.

### Extraction of total RNA and quantitative real-time PCR

Total RNA was isolated from the kidney tissues using TRIzol (Invitrogen; Thermo Fisher Scientific, Inc., Waltham, MA). Reverse transcription was conducted using a TaqMan advanced miRNA cDNA synthesis kit (A28007; Applied Biosystems, Foster City, CA). The qPCR was carried out in the TaqMan miRNA assay kit (4440887; Applied Biosystems). The U6 functioned as the internal control. In this experiment, we performed the Taqman-based real-time PCR and the probe of mmu-miR-122-5p/U6 was purchased from Applied Biosystems (catalog number: 002245/001973). All PCR data were analyzed by the LightCycler 96 SW 1.1 software, and each sample was shown as 2^−ΔΔCt^ values.

### Cell culture

The Boston University mouse proximal tubular cell line (BUMPT) was used in this study, and the cells were cultured in medium as described in other studies [33]. To establish the diabetic cell model, the cells were treated with 35 mM glucose for 24 h. Control cells were maintained in normal medium. In some experiments, 200 nM microRNA mimic, HIF-1α plasmid or NC oligonucleotides were transfected into BUMPT cells with Lipofectamine 2000 following the manufacturer’s instructions

### Cell immunofluorescence

Cells were grown on coverslips, washed three times with PBS, fixed in 4% paraformaldehyde for 20 min, permeabilized with 0.1% Triton X-100, and then incubated in blocking buffer. The cells were subsequently incubated with FIH-1 antibody (dilution: 1:200) overnight. Then, the cells were incubated with FITC-conjugated secondary antibodies (dilution: 1:500) and examined with a fluorescence microscope and the representative figures were exhibited.

### Western blot analysis

Cells or kidney tissues were lysed with 2% SDS buffer containing protease inhibitor cocktail (Sigma-Aldrich, P8340). The equal amount of proteins from different samples was separated on SDS-polyacrylamide gels. After being transferred onto polyvinylidene difluoride membrane, membrane was incubated with 5% fat-free milk to reduce unspecific signals and probed subsequently with primary antibodies (dilution: 1:1000) and horseradish peroxidase-conjugated secondary antibodies (dilution: 1:5000). Antigen-antibody complex was visualized with an enhanced chemiluminescence kit (Thermo Fisher Scientific, 32106).

### Luciferase microRNA target reporter assay

The 3′-UTR of the mouse FIH-1 gene inserted into the 3′-UTR of the luciferase gene in the pMIR-REPORT luciferase plasmid. The plasmids with or without the insert were co-transfected with pMIR-REPORT β-gal control plasmid and 200 nM miR-122-5p mimics into BUMPT cells. One day after the transfection, the lysate was collected in reporter lysis buffer from the Luciferase Assay System (Promega, Madison, WI). The luciferase activity was normalized with β-galactosidase activity. The ratio of the normalized value between miR-122-5p and NC group was used for comparison.

### Statistical analysis

Student’s *t*-test was used to show the significant difference between two groups and ANOVA analysis was used for multi-group difference analysis. Data are expressed as the means ± SD. *p* < 0.05 was considered significant. GraphPad Prism 7.0 (GraphPad Software, La Jolla, CA) was used for all calculations.

## Results

### miR-122-5p is induced in renal tubules in DN mice

**Figure 1.**
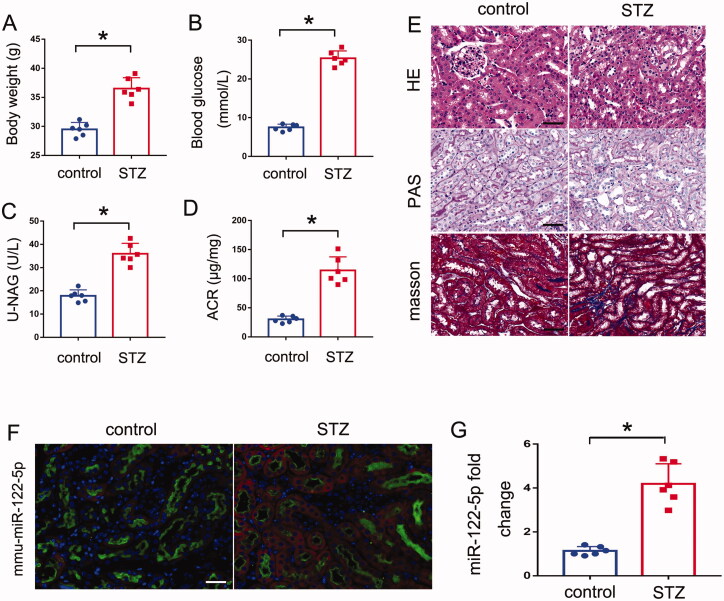
miR-122-5p is induced in renal tubules in DN mice. Eight-week-old C57BL/6J mice were injected with 50 mg/kg STZ for 5 consecutive days. Control mice were injected with normal saline. The mice were euthanized after 12 weeks. (A) body weight changes in control and STZ mice; (B) Blood glucose concentrations in each group; (C) urinary NAG levels; (D) urinary ACR levels; (E) representative images of hematoxylin–eosin (HE) staining, periodic acid-Schiff (PAS) and Masson staining, scale bar: 50 μm; (F) *in situ* hybridization showing miR-122-5p increase in kidney tissues after STZ treatment. Representative images of double immunostaining with miR-122-5p and proximal tubule marker, Lotus tetragonolobus lectin (LTL). Scale bar: 50 μm; (G) qPCR analysis of miR-122-5p in mouse kidneys with STZ treatment or normal saline. The level of miR-122-5p was normalized to the level of U6 (internal loading control) of the same samples to determine the ratio with the ratio of control mice arbitrarily set as 1.All the values are expressed as mean ± SD (*n* = 6), *p < 0.05.

To identify specific miRNAs involved in the pathogenesis of DN, we initially tested the mouse model of STZ treatment. As shown in Figure 1(A,B), both the body weights and blood glucose levels of STZ mice were higher than control mice. In addition, a significant increase in the urinary NAG and ACR was also observed in STZ mice compared to control mice (Figure 1(C,D)). Consistently, histological analysis by H&E, PAS and Masson staining also showed the most obvious tubular injury in STZ mice (Figure 1(E)). Briefly, In STZ group, PAS and HE staining showed notable morphological changes, including glomerular hypertrophy, increased mesangial matrix, and increased tubular epithelial disruption; Masson staining showed remarkable renal fibrosis, including glomerulosclerosis and interstitial fibrosis. These results suggest increased tubule injury in STZ induced DN mice. Then we collected kidney tissues for microarray analysis of miRNA expression (every group including 3 mice) and found a series of miRNAs with altered expression (Table 1). Among which, miR-122-5p was significantly induced. By real-time PCR, we further verified the induction of miR-122-5p in the kidneys of the STZ-treated mice as compared with the control mice (Figure 1(G)). *In situ* hybridization analysis, we conducted the double immunofluorescence staining using the proximal tubule marker LTL (Lotus tetragonolobus lectin) demonstrated miR-122-5p expression in renal proximal tubules (Figure 1(F))

**Table 1.** Microarray profiling of microRNA expression in DN

Up-regulated miRNAs		Down-regulated miRNAs	
miRNAs	Log_2_ fold changes	miRNAs	Log_2_ fold changes
miR-290-3p	8.4	miR-1668	10.2
miR-203-5p	7.5	miR-1906	10.1
miR-188-3p	7.4	miR-546	9.8
miR-122-5p	6.8	miR-697	9.5
miR-710	6.6	miR-709	7.9
miR-190b	6.5	miR-467f	7.3
miR-23a-5p	5.5	miR-670	7.3
miR-3965	5.4	miR-511-3p	6.8
miR-761	5.1	miR-212-3p	5.5
miR-145b	5.1	miR-1951	5.0

### miR-122-5p attenuates STZ-induced DN in mice

**Figure 2.**
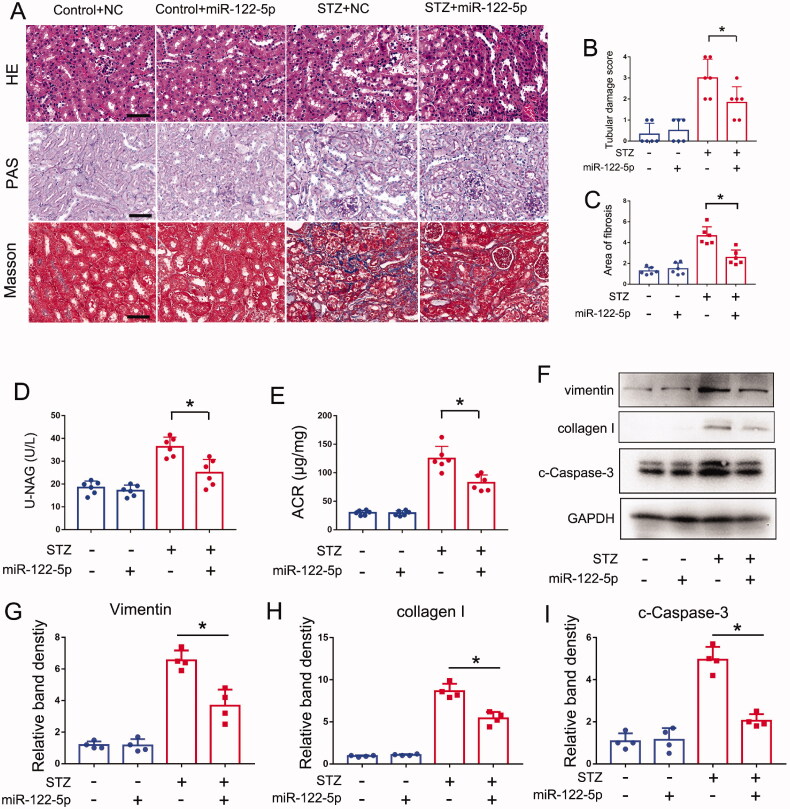
miR-122-5p attenuates STZ-induced DN in mice. miR-122-5p mimic (3mg/kg) or negative control (NC) were delivered to C57BL/6 mice through tail vein injection every 2 weeks following STZ injection. Kidney tissues were collected at 12 weeks after STZ injection. Control mice were injected with normal saline. (A) Pathological changes in kidneys were demonstrated by HE, PAS and Masson staining, scale bar: 50 μm; (B) the graph shows semiquantitative tubular injury scores, HE staining was used to assess the tubules damage score; (C) the graph shows semiquantitative renal fibrosis, Masson staining was used to assess the relative area of fibrosis; (D) urinary NAG levels; (E) urinary ACR levels; (F) expression of Vimentin, collagen I and cleaved caspase-3 were detected by Western blotting, GAPDH was used as internal control. (G–I) Quantification analysis of the related band intensity. All the values are expressed as mean ± SD (*n* = 6 for (B-E) and *n* = 4 for (G–I)), *p < 0.05.

How does the role of miR-122-5p in DN? In this regard, we tested the effects of miR-122-5p mimic in STZ induced DN mouse models. In control mice, the miR-122-5p mimic did not cause structural damages and renal fibrosis in kidneys, but it significantly attenuated the tubular damage and interstitial fibrosis in STZ mice (Figure 2(A–C)). Functionally, miR-122-5p mimic also obviously reduced the levels of urinary NAG and ACR upon STZ treatment (Figure 2(D,E)). In addition, our immunoblot analysis indicated that miR-122-5p mimic could reduce the levels of collagen I and vimentin (Figure 2(F–H)). Then, we detected tubular cell apoptosis by immunoblot of cleaved-caspase 3. As shown in Figure 2(F,I), immunoblot analysis also detected less cleaved caspase-3 in the kidney tissues of STZ + miR-122-5p-treated mice than in mice treated with STZ only. These findings support a protective role of miR-122-5p in DN.

### Inhibition of miR-122-5p exaggerates STZ-induced DN in mice

**Figure 3.**
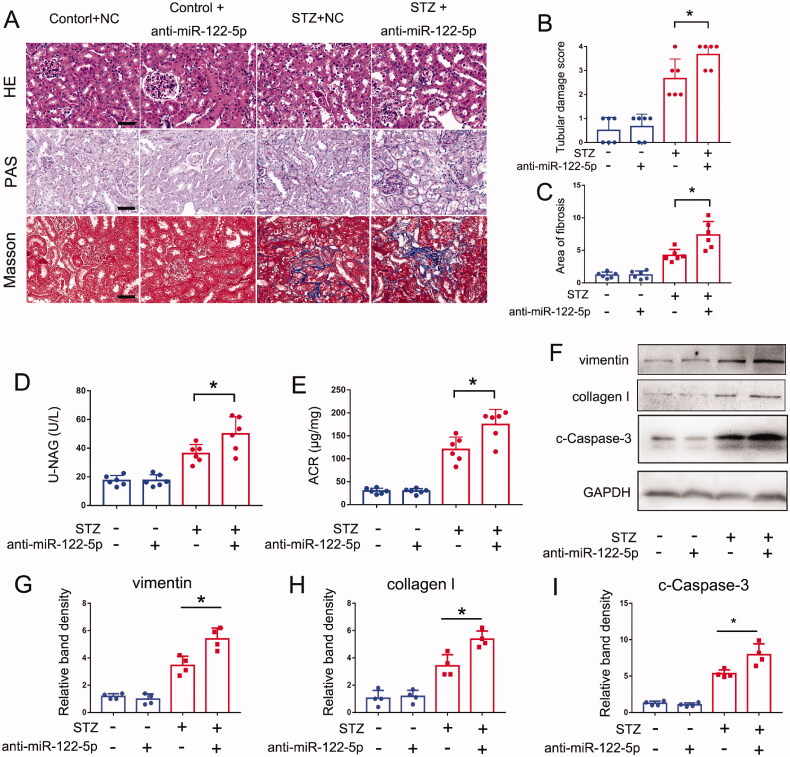
Inhibition of miR-122-5p exaggerates STZ-induced DN in mice. C57BL/6 mice were given 6 mg/kg anti-miR-122-5p or scrambled sequence oligonucleotides (NC) through tail vein injection every 2 weeks following STZ injection. Kidney tissues were collected at 12 weeks after STZ injection. Control mice were injected with normal saline. (A) Pathological changes in kidneys were demonstrated by HE, PAS and Masson staining, scale bar: 50 μm; (B) the graph shows semiquantitative tubular injury scores; HE staining was used to assess the tubules damage score; (C) the graph shows semiquantitative renal fibrosis, Masson staining was used to assess the relative area of fibrosis; (D) urinary NAG levels; (E) urinary ACR levels; (F) expression of vimentin, collagen I, cleaved caspase-3 were detected by Western blotting, GAPDH was used as internal control. (G–I) quantification analysis of the related band intensity. All the values are expressed as mean ± SD (*n* = 6 for (B–E) and *n* = 4 for (G–I)), *p < 0.05.

To further identify the role of miR-122-5p in DN, we detected the effect of anti-miR-122-5p in STZ induced DN mouse models. Locked nucleic acid-modified (LNA-modified) anti-miR-122-5p or scrambled sequence oligonucleotides (NC) were administered to mice. Similar to the results of miR-122-5p mimic, anti-miR-122-5p also caused neither tubular structural injury nor renal fibrosis in control mice, but it significantly increased the levels of urinary NAG and ACR and aggravated tubular injury and renal fibrosis in STZ-induced DN mice (Figure 3(A–E)). The immunoblot analysis showed that anti-miR-122-5p could promote the expression of collagen I and vimentin (Figure 3(F–H)). In addition, anti-miR-122-5p promoted cell apoptosis in this model, as shown by cleaved caspase-3 immunoblotting (Figure 3(F,I)). These findings further support the conclusion that miR-122-5p plays a protective role in DN.

### FIH-1 is a downstream target of miR-122-5p in DN

**Figure 4.**
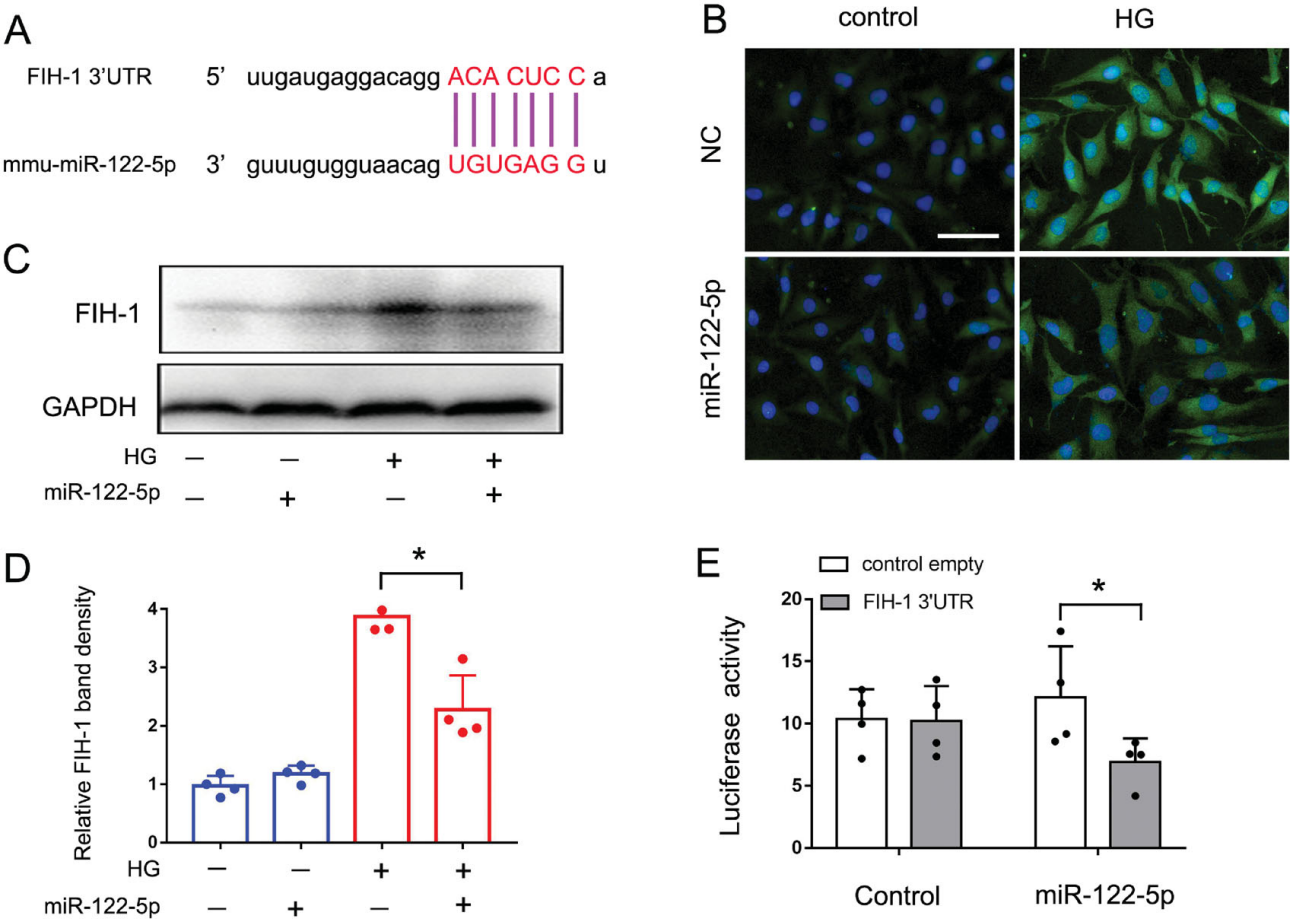
FIH-1 is a downstream target of miR-122-5p in DN. BUMPT cells were transfected with 200 nM miR-122-5p mimic or negative control (NC) for 24 h and then treated with high glucose (35 mM) for another 24 h. Control cells were maintained in normal medium. (A) The predicted miR-122-5p binding site in 3’UTR of FIH-1 mRNA; (B) immunofluorescence analysis showing the repressive effect of miR-122-5p on FIH-1 expression. Images were collected by laser scanned confocal microscopy, scale bar: 50 μm; (C) immunoblot analysis showing the repressive effect of miR-122-5p on FIH-1 expression. GAPDH was used as internal control. (D) Quantification analysis of the related band intensity. Values are expressed as mean ± SD (*n* = 4), *p < 0.05. (E) MicroRNA target reporter assay of FIH-1 3′-UTR. The putative miR-122-5p target sequence of the FIH-1 3′-UTR was cloned into the pMIR-REPORT vector. This and empty vector were transfected with miR-122-5p mimic or NC oligonucleotides to analyze luciferase activity. Values are expressed as Mean ± SD (*n* = 4), *p < 0.05.

To understand the mechanism whereby miR-122-5p contributes to DN, we investigated it downstream of target gene. By using online databases (PITA and Target Scan), we identified a conserved putative miR-122-5p targeting site in the 3’UTR of FIH-1 mRNA (Figure 4(A)). To determine whether FIH-1 is indeed a target of miR-122-5p, we first examined whether overexpression of miR-122-5p in BUMPT cells would affect FIH-1 expression. As shown in Figure 4(B–D), both of the immunofluorescence staining and immunoblot indicated the expression of FIH-1 was significantly repressed by the transfection of miR-122-5p mimics in high glucose-treated BUMPT cells. Further, to determine if FIH-1 is a direct target of miR-122-5p, we prepared a microRNA Luciferase reporter. The FIH-1 3’UTR constructs or empty vectors were transfected into BUMPT cells along with miR-122-5p mimic or negative control oligo (NC). miR-122-5p mimic inhibited the luciferase activity in FIH-1 3’UTR-miR-122-5p transfected cells, whereas the negative control oligo did not (Figure 4(E)). Taking all together, these results indicate FIH-1 is a direct target of miR-122-5p.

### miR-122-5p attenuates high glucose-induced tubular injury through targeting FIH-1

**Figure 5.**
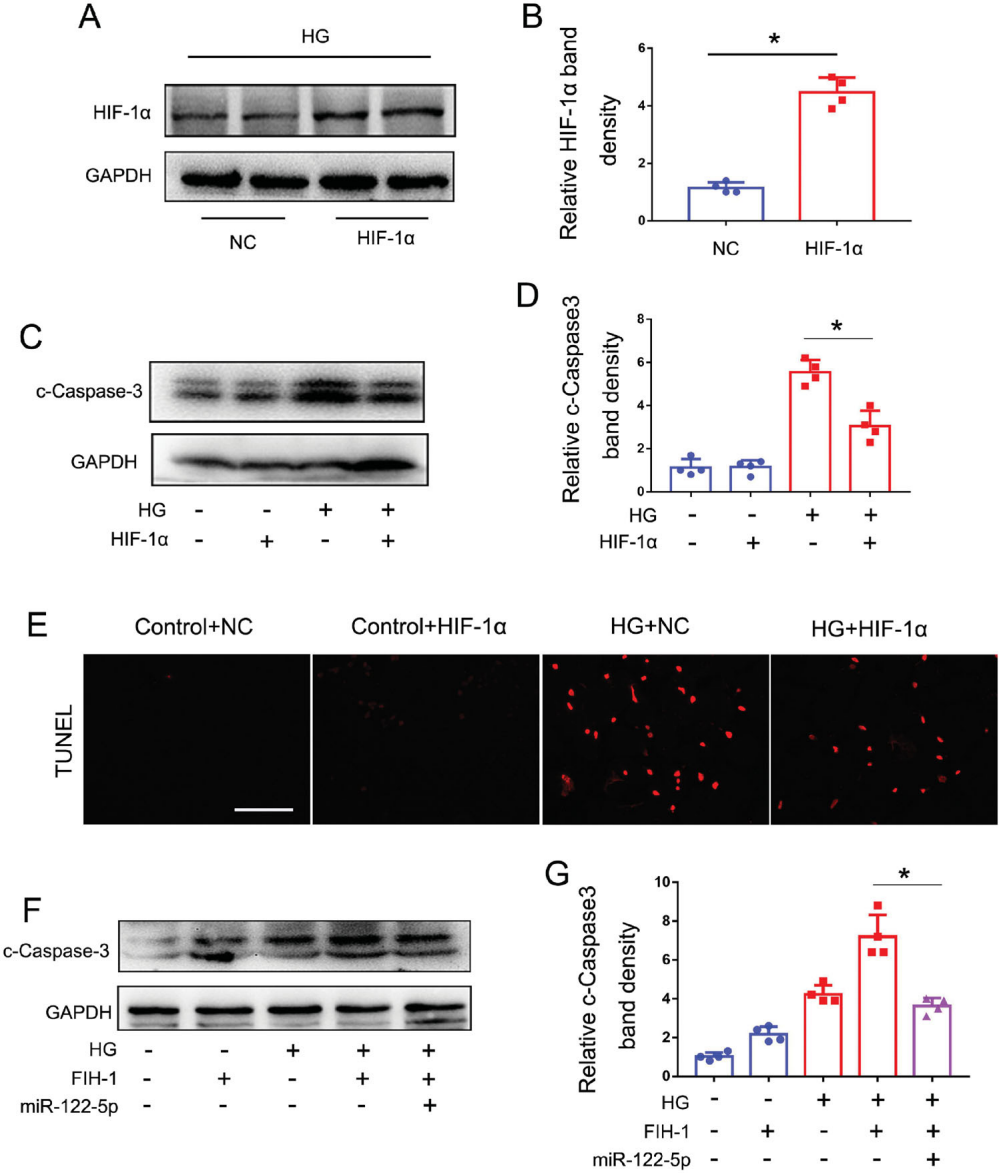
miR-122-5p attenuates high glucose-induced tubular injury through targeting FIH-1. BUMPT cells were transfected with pHIF-1α plasmid, FIH-1 plasmid, or negative control (NC) for 24 h and then treated with high glucose (35 mM) for another 24 h. Control cells were maintained in normal medium. (A) Immunoblot of HIF-1α to confirm the successful delivery of pHIF-1α plasmid; (B) quantification analysis of the related band intensity. Values are expressed as Mean ± SD (*n* = 4), *p < 0.05. (C) Immunoblot of cleaved-caspase 3 from BUMPT cells with negative control (NC) or HIF-1α plasmid transfection. GAPDH was used as loading controls. (D) Quantification analysis of the related band intensity. Values are expressed as mean ± SD (*n* = 4), *p < 0.05. (E) Representative images of TUNEL staining, scale bar: 100 μm; (F) immunoblot of cleaved-caspase 3, GAPDH was used as loading controls. (G) Quantification analysis of the related band intensity. Values are expressed as mean ± SD (*n* = 4), *p < 0.05.

It has been indicated that FIH-1 could inhibit the activity of hypoxia-inducible factor 1 (HIF-1) by preventing HIF-1α from binding to p300/CBP [34]. Thus, to determine the role of FIH-1 in tubule injury in DN, we examined the effects of HIF-1α overexpression on apoptosis in high glucose-treated BUMPT cells. First, we confirmed the successfully delivery of HIF-1α plasmid through WB analysis (Figure 5(A,B)). Then, we found HIF-1α-overexpression cells had lower levels of cleaved caspase-3 activation upon HG treatment, indicative of less apoptosis (Figure 5(C,D)). Our TUNEL results also indicated that high glucose induced less cell death in HIF-1α-overexpression cells than in the cells transfected with control sequences (Figure 5(E)). Further, we found FIH-1-overexpression cells had higher levels of cleaved caspase-3 activation upon HG treatment, indicative of more apoptosis. However, miR-122-5p transfection could partly reverse these changes and reduce apoptosis (Figure 5(F,G)). Collectively, these results indicated that miR-122-5p could attenuate tubular cell apoptosis in DN through targeting FIH-1.

## Discussion

Recently, increasing evidence showed that miRNAs have been implicated in the pathogenies of DN. Therefore, there is a need for further studies to determine the role of miRNAs in the regulation of DN with the hope of discovering new therapies for DN [35]. In this study, we report the following major findings: (1) miR-122-5p is significantly induced in renal tubular cells in STZ induced DN mouse models; (2) functionally, miR-122-5p attenuates tubular injury and cell apoptosis, indicating that miR-122-5p induction in DN is an adaptive or protective mechanism; (3) mechanistically, miR-122-5p directly targets and inhibits FIH-1 expression to enhance the HIF-1α activity, finally ameliorating tubular injury in DN. Together, these findings unveil a novel miR-122-5p/FIH-1/HIF-1α pathway which can delay the DN progression. Essentially, miR-122-5p is induced in DN, leading to the suppression of its target gene FIH-1. The decrease of FIH-1 enhances the activity of HIF-1α, which finally delay the progress of DN, providing an intrinsic protective mechanism.

Diabetes is a common chronic metabolic disease which has affected about half a billion people in the world and DN affects approximately one-third of diabetes patients. Thus, DN is not only a health crisis but also a global social disaster [36]. However, the specific molecular mechanism of DN remains unclear, and there is still a lack of effective therapies. Tubular injury is widely recognized to be associated with the pathogenesis of DN. Thus, studies on new targets for tubular injury are necessary for exploring prospective therapies for DN treatment [37]. It has been reported that miRNAs have been implicated in the pathogenesis of DN, including renal tubular epithelial cell injury [36]. For example, Li et al. found that miR-25 inhibited high glucose-induced apoptosis in renal tubular epithelial cells *via* PTEN/AKT pathway [38]. Based on these findings, specific miRNAs may become new therapeutic target for DN. In the present study, we have indicated that miR-122-5p is significantly induced in kidney tubular cells in DN. Moreover, inhibition of miR-122-5p led to increased cell death and serve tubular injury and promoted DN progression following STZ treatment in mice, whereas supplementation of miR-122-5p mimic had kidney protective effects in this model. These results indicate a protective role of miR-122-5p in DN. Accordingly, miR-122-5p up-regulation in DN is an adaptive or protective response in this disease condition. Of course, there are a few important clinical studies, which are highly relevant to the miR-122-5p [39–42]. For example, a recent study from Regmi and colleagues showed that serum levels of miR-122-5p were positively associated with FBG, HbA1c and, importantly, with urine albumin, and negatively associated with eGFR. These results seem to contrast the concept that miR-122-5p protects renal function in patients with diabetes [41]. However, this study did not detect the role of miR-122-5p in DN. In fact, whether miR-122-5p is protective or injurious to the kidney, it can rise in the serum or urine. For example, miR-494 was increased in the serum and urine during acute kidney injury in patients, however, it can promote ischemic AKI through targeting ATF3 [43]. In another study, miR-668 was significantly increased in urine in patients with acute kidney injury, and it can protect kidney through targeting MTP18 [44].

How does miR-122-5p contribute to DN? To address this, our *in vitro* studies identified miR-122-5p could combine with FIH-1 3′ UTR and negatively regulated the FIH-1 protein levels, indicating FIH-1 as a direct target of miR-122-5p (Figure 4). FIH-1 is an asparagine hydroxylase that interacts with hypoxia-inducible factor 1α (HIF-1α) to regulate transcriptional activity of HIF-1. Generally, FIH-1 inhibits the activity of HIF-1 by preventing HIF-1α from binding to p300/CBP [34,45]. Recently, it has been indicated that FIH-1 played a crucial role in the pathogenesis of CKD through targeting HIF-1α [46]. It is known that HIF-1 is a critical molecule for mitigating hypoxia-induced damage and exists as a heterodimer comprising two subunits: a variable α-subunit and a constitutively expressed β-subunit. In the kidney, HIF-1α is expressed by most renal tubular epithelial cells [47,48]. Increasing studied have shown that an oxygen deficit is present in DN and that enhancing HIF-1 signaling ameliorates the progression of DN [49,50]. For example, a study conducted by Jiang et al. have indicated that HIF-1α could ameliorate tubular injury in DN *via* HO-1-mediated control of mitochondrial dynamics [51]. In our present study, overexpression of HIF-1α by transfection of HIF-1α plasmid reduced tubular cell death during high-glucose treatment, suggesting a protective role of HIF-1α in DN (Figure 5). Taking together, these findings unveil a novel miR-122-5p/FIH-1/HIF-1α pathway which can attenuate the DN progression. Essentially, miR-122-5p is induced in DN, leading to the suppression of its target gene FIH-1. The decrease of FIH-1 enhances the activity of HIF-1α, which finally delay the progress of DN, providing an intrinsic protective mechanism.

In conclusion, the present study demonstrated an induction of miR-122-5p in diabetic nephropathy and further investigated its biological function and molecular mechanisms. All the experimental results indicated that miR-122-5p ameliorated tubular injury and delayed the progression of DN by targeting FIH-1/HIF-1α signaling, which may provide a potential diagnostic or therapeutic target for DN.

## Data Availability

The data that support the findings of this study are available from the corresponding author, [LL], upon reasonable request.
